# Comparison of Particle-Associated Bacteria from a Drinking Water Treatment Plant and Distribution Reservoirs with Different Water Sources

**DOI:** 10.1038/srep20367

**Published:** 2016-02-02

**Authors:** G. Liu, F. Q. Ling, E. J. van der Mark, X. D. Zhang, A. Knezev, J. Q. J. C. Verberk, W. G. J. van der Meer, G. J. Medema, W. T. Liu, J. C. van Dijk

**Affiliations:** 1 Sanitary Engineering, Department of Water Management, Faculty of Civil Engineering and Geosciences, Delft University of Technology, P.O. Box 5048, 2600 GA Delft, the Netherlands; 2 Oasen Water Company, PO BOX 122, 2800 AC, Gouda, the Netherlands; 3 Department of Civil and Environmental Engineering, University of Illinois Urbana-Champaign, 205 N. Mathews Ave., Urbana, Illinois 61801, U.S.A; 4 Dunea Water Company, P.O. Box 756, 2700 AT Zoetermeer, the Netherlands; 5 Het Water Laboratorium, P.O. Box 734, 2003 RS Haarlem, the Netherlands; 6 KWR Watercycle Research Institute, P.O. Box 1072, 3430 BB Nieuwegein, the Netherlands

## Abstract

This study assessed the characteristics of and changes in the suspended particles and the associated bacteria in an unchlorinated drinking water distribution system and its reservoirs with different water sources. The results show that particle-associated bacteria (PAB) were present at a level of 0.8–4.5 × 10^3^ cells ml^−1^ with a biological activity of 0.01–0.04 ng l^−1^ ATP. Different PAB communities in the waters produced from different sources were revealed by a 16S rRNA-based pyrosequencing analysis. The quantified biomass underestimation due to the multiple cells attached per particle was ≥ 85%. The distribution of the biologically stable water increased the number of cells per particle (from 48 to 90) but had minor effects on the PAB community. Significant changes were observed at the mixing reservoir. Our results show the characteristics of and changes in suspended PAB during distribution, and highlight the significance of suspended PAB in the distribution system, because suspended PAB can lead to a considerable underestimation of biomass, and because they exist as biofilm, which has a greater mobility than pipe-wall biofilm and therefore presents a greater risk, given the higher probability that it will reach the customers’ taps and be ingested.

The bacteria are present in different phases, or locations, in drinking water distribution systems, where they can grow and multiply^1^,^2^. These phases are: the bulk water (planktonic bacteria that flow through the water main); pipe-wall biofilm (biofilm bacteria that attach to the pipe surface); suspended solids (particle-associated bacteria, or particulate matter, suspended in the water and transported throughout the network); and loose deposits (particle associated bacteria, or particulate matter that is accumulated/retained in the distribution pipes)^1^,^2^,^3^. Photographic images taken in an operational distribution pipe illustrate the four phases (Fig. S1). These phases are dynamically interrelated: depending on the local hydraulic conditions, the loose deposits and pipe-wall biofilm may be resuspended and detached to become suspended solids and reach the customers’ taps, or the suspended solids may settle and accumulate in the distribution pipes as loose deposits^1^,^2^,^3^.

The particles in drinking water distribution systems have primarily been studied in terms of their physiochemical aspects^4^,^5^,^6^. Only a few studies have examined the organic components of the loose deposits^7^,^8^,^9^,^10^. Although only limited information on the microbial composition of suspended/settled particles has been reported, researchers have found a considerable amount of bacteria associated with loose deposits, including *Mycobacteria* spp^10^.

Typically, planktonic bacteria (PB) are subject to rapid washout together with bulk water, due to the plug flow conditions^1^,^11^ and to the fact that their size ( < 1 μm) is insufficient for them to settle as a deposit^12^; in contrast, particle-associated bacteria (PAB) can settle and accumulate in the DWDS as loose deposits^2^,^7^,^8^,^13^. The accumulated PAB may be transferred into the bulk water by hydraulic peaks—e.g., during morning peaks of water usage, pipe bursts, and firefighting operations—resulting in cell peaks at the taps^2^. Compared to the bacteria harbored by pipe-wall biofilm in the DWDS, PAB present an even higher risk because their greater mobility increases the chance that they will reach the customers’ taps and be ingested^2^,^14^.

The significance of PAB in drinking water is also related to their higher resistance to disinfection compared to^15^,^16^ PB^13^,^14^. Moreover, PAB have been considered to be the seeds for regrowth downstream^17^. The presence of PAB also introduces the potential for an underestimation of the bacterial numbers because, regardless of the number of cells attached to one particle, they will either not be counted or be counted as one cell by the currently used cell enumeration methods, e.g., heterotrophic plate counts (HPC) and flow cytometry cell counting^3^,^17^,^18^,^19^.

In a previous study we reported the quantification and identification of particle-associated bacteria in unchlorinated treated water^3^; PAB concentrations of 1.0–3.5 × 10^3^ cells ml^−1^ and 0.04–0.154 ng l^−1^ATP were found in the unchlorinated treated water from three Dutch treatment plants. Multiple cells per particle were confirmed, and on average 25–50 cells were attached to a single particle (ranging from 1–100 μm but mostly from 1–2 μm). A community study using pyrosequencing has revealed that the members of the *Proteobacteria* dominated in all of the sampled PAB communities, followed by *OP3* and *Nitrospirae*. However, the study only sampled the treated water from treatment plants; the information regarding the PAB in DWDSs remains unknown.

Hence, there is a clear need to explore the suspended PAB in DWDSs. The primary objective of this study was to investigate the suspended PAB in an unchlorinated drinking water system. It was conducted to explore the characteristics of and changes in suspended PAB in drinking a water treatment plant and distribution reservoirs with two different water sources.

## Results

### Physiochemical characterization of the suspended particles

**Figure 1:**
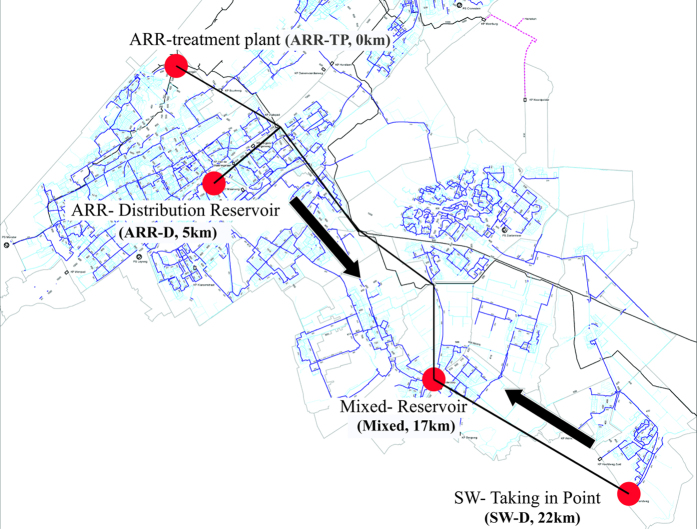
Schematic diagram of the sampling sites in the distribution system, showing the artificial recharge and recovery (ARR) water treatment plant (ARR-TP), the ARR-Distribution Reservoir (ARR-D), the Mixed-Reservoir (Mixed), and the connection point that takes in the treated surface water (SW-D). The map was made from the digital map of distribution pipe network of the studied supply area. The digital map was provided freely by the water company (www.dunea.nl); the sampling points and location information was added and edited using Adobe Illustrator.

**Figure 2:**
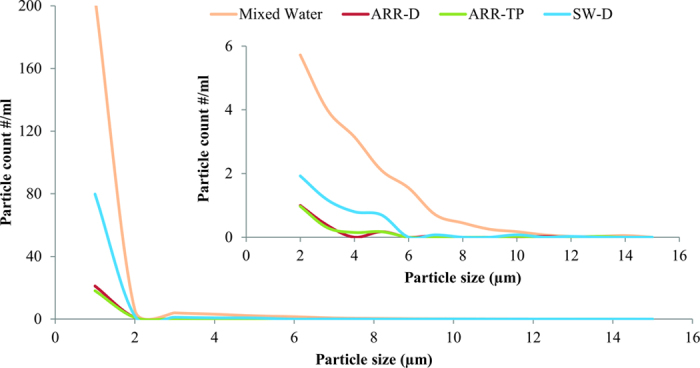
The particle-size distribution in the water samples at different sampling sites. The main figure shows the size distribution of particles ≥ 1 μm, and the inside figure shows the size distribution of particles ≥ 2 μm.

PAB were collected from four locations in the distribution system and analyzed to investigate their characteristics and changes in an unchlorinated drinking water distribution system (Fig. 1). As shown in Table S1, the particle number in the treated (ARR-TP) and distributed (ARR-D) artificial recharge and recovery water was lower than in the distributed surface water (SW-D). Regarding the particle sizes, Fig. 2 shows that the particles at ARR-TP and ARR-D were smaller than those at SW-D. A significant increase in the particle load was observed in the water samples from the mixing reservoir (Mixed). Spherical-like particles were found in the treated and distributed ARR water, whereas thin, layer-like particles were found in the distributed surface water. The elemental analysis showed (Fig. S3) that the particles primarily consisted of carbon (C), oxygen (O), silicon (Si), sodium (Na), potassium (K), calcium (Ca), and iron (Fe). Fe was not found in ARR water. The Fe in the samples collected at the mixing reservoir (Mixed) was higher than that in the distributed surface water (SW-D). The carbon content was lower in the ARR water than in the surface water, whereas the carbon content at the mixing reservoir was between that of the ARR water and the surface water.

### Quantification of the PAB

**Figure 3:**
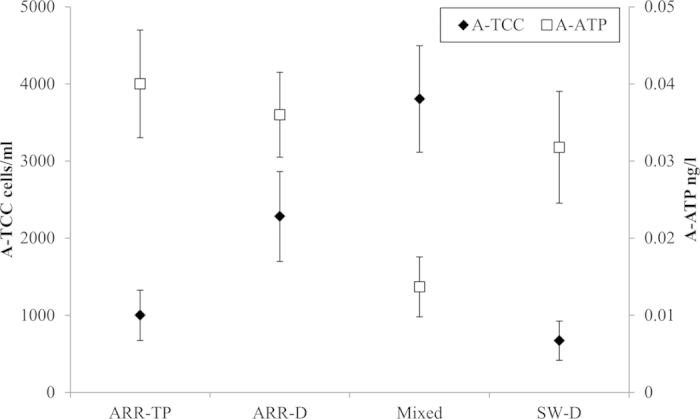
Average attached TCC (A-TCC) and attached ATP (A-ATP) results of the PAB before and after mixing in the distribution system (n = 3).

In the unchlorinated drinking water network, we found 1.0–4.0 × 10^3^ cells ml^−1^ and 0.01–0.05 ng l^−1^ ATP of PAB. Comparable amounts of PAB were found at ARR-TP and SW-D (Fig. 3). The distribution of the ARR water led to an increase in the cell numbers attached to the particles, whereas the change in the bacterial activities was insignificant. At the mixing reservoir (Mixed water), the cell numbers of PAB were higher, whereas the bacterial activities were lower than at the other locations. The cells per particle were calculated by Equation (1) (Table S1). On average, 48 cells per particle were found at ARR-TP. The ARR-D water contained 91 cells per particle. There were only 7 cells per particle in the SW-D water. At the mixing reservoir (Mixed water), 16 cells were attached to a single particle.

### Identification of the PAB

We obtained 2988, 6377, 22496 and 10423 bacterial 16S rRNA gene sequences from PAB sampled from ARR-TP, ARR-D, SW-D, and Mixed water, respectively. The observed OTUs and the Chao1 and Shannon indices are shown in Table S2. Rarefaction analysis results are shown in Fig. S2. At the mixing reservoir, an increase was observed in the bacterial diversity.

### Community composition

**Figure 4:**
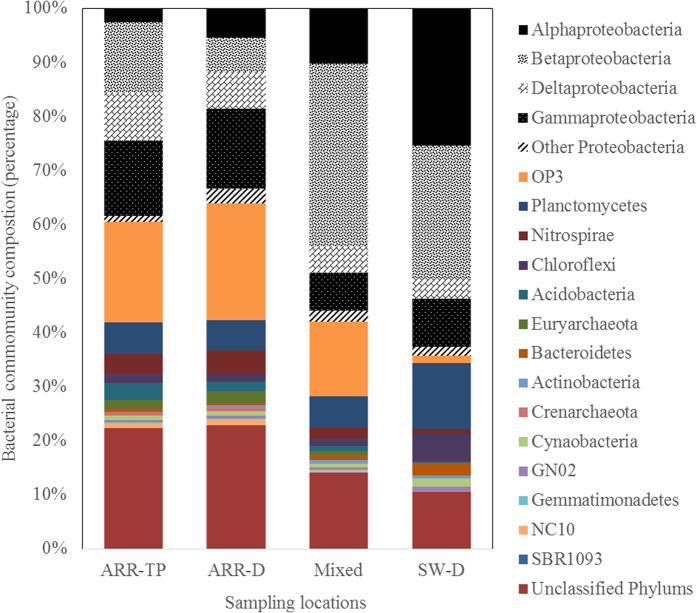
Taxonomic assignment of the 16 s rRNA gene sequences retrieved from the PAB samples, classified by phylum, with the phylum of *Proteobacteria* shown in the subclasses of *Alphaproteobacteria, Betaproteobacteria, Deltaproteobacteria, Gammaproteobacteria*, and other *Proteobacteria* (shown in black in the upper part of the figure).

In the treated and distributed ARR water, fifteen phyla were detected. In the distributed ARR water (ARR-D, Fig. 4), *Proteobacteria* represented 36% of the total OTUs followed by the phyla of *OP3* (22%), *Planctomycetes* (6%), *Nitrospirae* (4%), *Acidobacteria* (2%), *Chloroflexi* (1%), and *Euryarchaeota* (2%). The remaining 8 phyla accounted for 4% of the total OTUs, and the unclassified phyla accounted for 22% of the total OTUs. Among the *Proteobacteria*, the subclasses of *Alphaproteobacteria*, *Betaproteobacteria*, *Deltaproteobacteria*, and *Gammaproteobacteria* constituted (on average) 6%, 6%, 7%, and 15% of the total OTUs, respectively. Compared with the bacterial community composition of the treated ARR water at the treatment plant (ARR-TP, previously reported^3^), only minor changes were observed at ARR-D. These slight changes during the distribution of the ARR water (ARR-TP to ARR-D) included a slight decrease in the *Proteobacteria* percentage from 39% to 36%, whereas the *OP3* percentage increased from 19% to 22%. Within the *Proteobacteria*, the *Alphaproteobacteria* increased from 3% to 6%, *Betaproteobacteria* and *Deltaproteobacteria* decreased, respectively, from 13% to 6%, and from 9% to 7%.

In the samples at the mixing reservoir, 15 phyla types were detected (Fig. 4, Mixed). However, the bacterial community was different from that in the ARR water. The community was dominated by *Proteobacteria* followed by the phyla of *OP3* (14%), *Planctomycetes* (6%), *Nitrospirae* (2%), *Chloroflexi* (1%), and *Bacteroidetes* (1%). The remaining 9 phyla accounted for less than 4% of the total OTUs, and the unclassified phyla for 14%. Among the *Proteobacteria*, the subclasses of *Alphaproteobacteria*, *Betaproteobacteria*, *Deltaproteobacteria,* and *Gammaproteobacteria* accounted on average for 10%, 34%, 5%, and 7%, respectively.

Thirteen phyla types were detected, and a completely different bacterial community composition was found in the distributed surface water (Fig. 4, SW-D). The surface water bacterial community was dominated by *Proteobacteria* (65%), which was nearly double its percentage in the ARR water. The percentages of the phyla *Planctomycetes* (12%), *Chloroflexi* (5%), *Bacteroidetes* (2%), and *Cyanobacteria* (1%) were also more than two times higher than those in the ARR water. However, *OP3* and *Nitrospirae* decreased to 1%, and *Euryarchaeota* and *NC10* were undetected. The remaining 5 phyla accounted for less than 2% of the total OTUs, and the unclassified phyla accounted for 11%. Among the *Proteobacteria*, the subclasses of *Alphaproteobacteria*, *Betaproteobacteria*, *Deltaproteobacteria,* and *Gammaproteobacteria*, accounted on average for 25%, 24%, 4%, and 9% of the total OTUs, respectively. Thus, the percentages of *Alphaproteobacteria* and *Betaproteobacteria* were significantly higher than in the ARR water, whereas the percentages of *Deltaproteobacteria* and *Gammaproteobacteria* were slightly lower than in the ARR water.

A total of 54, 59, 68, and 58 genera were found at the ARR-TP, ARR-D, Mixed, and SW-D, respectively. The genera accounting for more than 1% are shown in Table S3. *Limnobacter* spp., *Caldilinea* spp., *CandidatusOdyssell* spp., *Rhodopirellula* spp., *Gallionella* spp., *Polynucleobacter* spp., and an unclassified genus of the *Oxalobacteraceae* order were detected in all cases, with the exception of the treated ARR water. *LCP-6* of *Thermodesulfovibrionaceae*, an unclassified genus of *Methanoscarinaceae*, and 5 genera of *OP3* were detected in all cases, with the exception of the distributed surface water.

### Principal coordinate analysis of the community similarity

**Figure 5:**
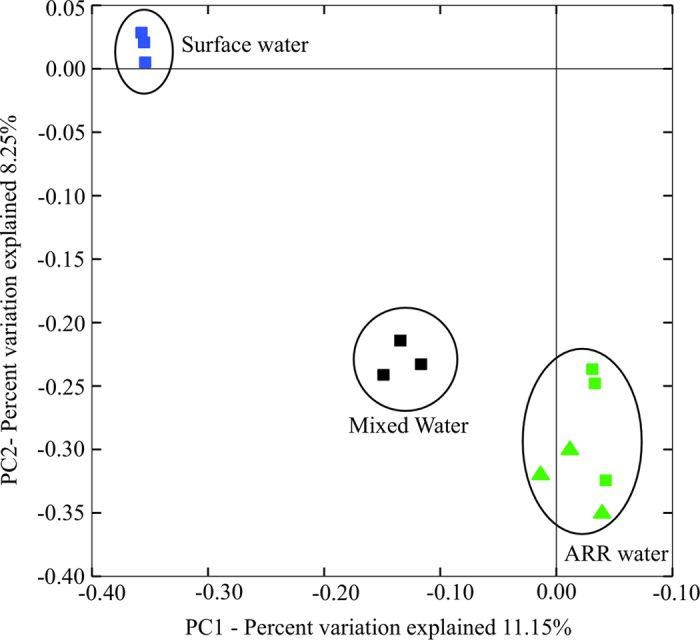
PCoA plot generated for all of the sampling locations. The results of the treated (triangle) and distributed (square) ARR water are shown in green; the results of the distributed drinking water produced from the surface water are shown in blue; and the results of the mixed water are shown in black.

Figure 5 shows the bacterial community similarity according to the PCoA plot. The results show that the ARR water and the surface water had a noticeably different bacterial community. Additionally, they show that the distribution of the ARR water did not influence the PAB community. The PAB in the water at the mixing reservoir had a new bacterial community cluster, which was between that of the treated ARR water and of the surface water.

## Discussion

To the best of our knowledge, this is the first study to investigate PAB characteristics and changes in a drinking water distribution system. The observed physiochemical characteristics of the particles are consistent with previous observations^3^,^4^,^6^,^13^,^14^,^20^,^21^. A previous study of ours covered several Dutch treatment plants including the plant that is the subject of this study. In contrast to the previous study’s results, we found a more complex elemental composition in the distribution system in this plant (ARR-TP). This may be due to particle aggregation/generation during distribution, such as corrosion (Fe), precipitation/flocculation (Al, Ca, Na, and K), bio-aggregation (C), and biofilm detachment (C, Na, and K)^3^.

The detection levels of more than 90 cells per particle were higher than the previously reported values^3^. This result further challenges the use of traditional bacteria enumeration methods for water quality monitoring which are employed in the current regulations, and in which the 90 cells would be counted as one. In addition, the consumers’ bacterial ingestion might be considerably underestimated, especially when the particle loads are high, such as the loads of ≥1.2 × 10^3^ particles per ml, which were detected at the customers’ taps in the distribution area of the ARR-D reservoir (results not shown). Therefore, the total PAB can be calculated, using the value measured at ARR-D: (90 cells per particle) × (1.2 × 10^3^ particles per ml), that is, ≥1.1 × 10^5^ cells ml^−1^. In fact, the PAB level could be even higher, because of the additional particles resulting from the resuspension of loose deposits, which constitute a reservoir for bacteria^7^,^8^,^14^. This underestimated value is comparable to the measured cell concentrations in the bulk water samples from the monitored customer tap (1.3 × 10^5^ cells ml^−1^) and is even higher than that detected in the water samples collected from the treatment plant (Table S1). According to Equation (2), the underestimation is ≥85% at the customers’ taps, which challenges the use of quantitative methods such as HPC in the current drinking water regulations.

The bacterial community composition was consistent with the previous studies on the drinking water bacteriology^22^,^23^,^24^,^25^ and with our recent study on PAB in treatment plants^3^; in all cases the *Proteobacteria* was the dominant phylum. Clear differences however were observed between the ARR water and the surface water, e.g., a high percentage of *OP3* was detected in the ARR water but not in the surface water. The candidate division *OP3* was found to thrive in anoxic environments^3^,^26^. Although both the systems use surface water as their source water, the ARR water treatment system involves a natural dune filtration process. The observed difference may be caused by the presence of the anoxic conditions in the ARR dune area, which is suitable for the *OP3* bacteria.

At the genus level, at the sampling locations of the distributed surface water and the water from the mixing reservoir where higher concentrations of iron were detected, a correspondingly higher percentage of iron bacteria were found (e.g., *Gallionella* accounted for 1% and *Crenothrix* accounted for 10%, Table S3). This difference may be because the treated surface water may favor the growth of iron bacteria; it has been reported that there are more iron-reducing and iron-oxidizing bacteria in the biofilms formed in distribution systems supplied by treated surface water than those supplied by treated groundwater^27^.

Generally, the distribution of biologically stable water had minor effects on the PAB; for example, on the particle number and particle size, the elemental composition of the particles, and the bacterial communities. The minor effects have been previously observed and reported on planktonic bacteria, e.g., Lautenschlager *et al.* have reported that biologically stable water can maintain a stable planktonic bacterial community during distribution^23^. This applies also to PAB, e.g., we have observed the strong similarity of the suspended PAB community sampled from three locations in another Dutch distribution system^14^. The stability of PAB may be due to the combination of the short retention time, the high and stable water quality, and the robust distribution system.

Nevertheless, the distribution of the ARR water involved a clear increase in the parameter of cells per particle, whereas the particle number remained stable. This increase may be caused by the multiplication of the attached cells and/or by the attachment of new cells to the particles during distribution. Because the bioactivity remained stable (A-ATP, Fig. 3), it is likely that the increased number of small cells attached to the particles did not significantly contribute to the ATP concentration. Regardless, the suspended particles offered a mobile surface area for the cells to attach to and for the biofilm to form on^28^.

The water quality parameters, such as ATP, total phosphate (TP), and dissolved organic carbon (DOC), showed simple mixing effects, in which the values ranged between the two types of water before mixing (Table S1). The data of the suspended PAB offered additional insight into the changes detected at the mixing reservoir. As shown in Fig. 2, the particle load at the mixing reservoir was approximately 2.5 times higher than that in the distributed surface water (SW-D), and was approximately 10 times higher than that in the treated and distributed ARR water (ARR-TP and ARR-D). The larger size and higher number of particles in the water at the mixing reservoir offer more surface area for cells to attach to. This is confirmed by the increased number of attached cells detected at the mixing reservoir. However, the attached biological activity (A-ATP content) was decreased, which might be due to the sensitivity of the microbes to the circumstantial changes, which they require time to acclimate to^29^. The lower number of cells per single particle in mixed water indicate that the increased particles at the mixing reservoir may hardly contain biomass. It is expected that more attached cells (A-TCC, biomass underestimation) will be found in the subsequent distribution system because of the high particle load with a greater available surface area for cells to attach to and grow on.

At the mixing reservoir, an increase in the bacterial diversity was found according to the parameters of the observed OTUs, the Chao1, and Shannon indices. These changes may be caused by the physiochemical changes of the particles (Fig. S3), because the increase of particle load and physiochemical characteristic changes may create new niches and subsequently influence the bacterial diversity^30^.

Most of the detected phyla (subclass of *Proteobacteria*) percentages showed moderating effects (changing to values between those for the ARR water and the surface water before mixing). For example, the percentage of *Alphaproteobacteria* (10%) at the mixing reservoir was higher than that in the ARR water (6%) but lower than that in the surface water (25%). The same effect was observed for *Deltaproteobacteria, OP3*, *Nitrospirae*, and *Euryarchaeota*. In addition to the simple moderating effects, the following three remarkable characteristics in the abundances were noted: 1) certain bacteria increased in abundance and were higher than their levels in both of the waters before mixing, e.g., *Betaproteobacteria* increased from 6–24% to 34%; 2) certain bacteria decreased in abundance and were lower than their levels in both of the waters before mixing, e.g., *Gammaproteobacteria* decreased from 10–14% to 7%; and 3) certain bacteria remained at the same abundances in one type of water, e.g., *Planctomycetes* was 6% at the mixing reservoir, which was the same level as in the treated and distributed ARR water.

As mentioned above in this study and reported elsewhere^23^, the distribution of biologically stable water only has a minor impact on the water’s bacterial community. This also applies in this case, in which a large-diameter pipe is used to transport water from the ARR-D (12 km) and the SW-D (5 km) to the mixing reservoir. Although the contributions of the pipelines (e.g., detachment of biofilms) and the mixing reservoir (e.g., disturbance of the reservoir sediments) cannot be completely excluded, it is likely that the mixing of two water types caused the differences and changes observed.

In summary, this study investigated the characteristics of and changes in PAB in DWDSs. Our results confirm the presence of multiple cells per particle, and suggest that the distribution of biological stable water has minor effects on the community of PAB. Moreover, this study demonstrates that the valuable information carried by PAB might be used to understand the processes occurring in DWDSs, such as detachment of biofilm or resuspension of loose deposits. This understanding can be achieved by comparing the finger-prints of PAB under normal and abnormal operational conditions, when problems arise, such as discoloration, sudden biomass peaks, or water meter clogging.

## Methods

### Description of the water treatment plant and the distribution system

The ARR water treatment plant obtains its source water from the Meuse River. The source water, after pre-treatment, is transported over 30 km to a dune area of natural lakes where it recharges the groundwater. After an average residence time of two months, the water is abstracted from the dunes. This abstracted artificial recharge and recovery (ARR) water is post-treated by softening, powdered activated carbon filtration, aeration, rapid sand filtration, and slow sand filtration before being pumped into the distribution system. Chlorination is avoided in the Netherlands.

The selected distribution system is supplied primarily by the treatment plant. At the end of the water supply network, near the boundary of a neighboring water company, there are several connection points which take in water. The neighboring treatment plant also obtains its surface source water (SW) from the Meuse River. After 5 months’ storage in the natural open reservoirs, the water is treated by passing it through fine sieves followed by flocculation, rapid sand filtration, UV disinfection, and activated carbon filtration; finally, a small amount of chlorine dioxide is dosed to decrease the colony counts resulting from the carbon filters. The two types of water are mixed in a reservoir in a ratio of 7:1 (ARR:SW) before it is supplied to customers in the area.

### Sampling

Four locations were selected (Fig. 1): 1) the ARR water treatment plant before the water is pumped into the distribution system (ARR-TP); 2) the distribution reservoir, which is fed only by water from the ARR water treatment plant (ARR-D); 3) the connection point on the 110 mm-diameter, PVC pipe, where water is taken in from the neighboring surface water treatment plant (distributed surface water, SW-D); and 4) the reservoir where the two types of water are mixed before being pumped into its supply area (Mixed). The general water quality data from each location are given in Table S1. The data at the ARR-TP had been previously reported and revealed the characteristics of the PAB in the treatment plants^3^. Those data were used in this study as a reference to compare and study any changes in the PAB in the distribution system.

The particle-associated bacteria were sampled and prepared as previously described^3^. Briefly, the PAB were pre-concentrated by filtering approximately 200 liters of water through glass fiber filters (Whatman, 1822–047, 1.2 μm). The choice of the filter pore size was explained in detail in the previous study^2^. Triplicate samples were obtained by running the multiple particle filtration systems (MuPFiS)^3^ on the same day of the week for three consecutive weeks for the PAB quantification (n = 3). For the pyrosequencing analysis, triplicate samples were obtained by one run of the MuPFiS (completed within one day for all of the locations, on the same day of the fourth week, n = 3). The filters with the pre-concentrated PAB were inverted and submerged into 5 ml of autoclaved tap water with glass beads immediately after filtration. All of the samples were maintained in a cooling box and transported to the laboratory within two hours after sampling. The bacteria were detached from the particles by low-energy ultrasonic treatment performed 3 times, for 2 minutes each (Branson ultrasonic water bath, 43 kHz). The obtained suspensions were used in the analyses.

### Analyses

The particle load was monitored by running particle counter (Met One, 32 channels, 1–100 μm) at each location for two weeks. The elemental composition of collected particles was analyzed by a JEOL JSM-840A scanning electron microscopy (SEM), coupled with secondary and backscattered electron detectors and an energy dispersive X-ray spectrometer. The PAB were quantified by Adenosine triphosphate (ATP) and total cell count (TCC) by flow cytometry. The ATP was measured as previously described^31^; the TCC was measured using a C6 flow cytometer (BD Accuri C6, United States), as described by Hammes *et al.*^32^.

The ATP and TCC results obtained for the PAB samples were defined as attached ATP (A-ATP) and attached TCC (A-TCC). Based on the ATP, TCC, and particle count results, the number of cells per particle was calculated according to the following equation (1), as previously described by Liu *et al.*^3^:


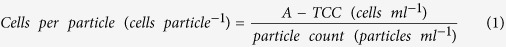


Based on the cells per particle and the particle number detected in the distribution system, the potential underestimation of the total cell number was calculated using the following equation (2):


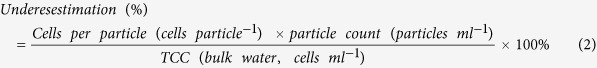


### 454 Pyrosequencing

The DNA was extracted from the suspension using the FastDNA Spin Kit for Soil (Q-Biogene/MP Biomedicals, Solon, OH, USA) according to the manufacturer’s instructions^33^,^34^, and was amplified with the bacterium-specific forward primer 27F and the reverse primer 534R^25^. The 454 pyrosequencing was performed with a 454 Life Sciences GS FLX series genome sequencer (Roche, Switzerland). The sequences were trimmed (resulting in an average sequence length of 230 bp). The merged alignments of the sequences aligned via the infernal aligner from the Ribosomal Database Project (RDP) pyrosequencing pipeline (http://pyro.cme.msu.edu/), and the NAST alignment tool from Greengenes was obtained via the software developed by the Biotechnology Center at the University of Illinois (UI) (http://acai.igb.uiuc.edu/bio/merge-nast-infernal.html). The RDP Classifier was used for the taxonomical assignments of the aligned 454 pyrosequences at the 97% confidence level. The total PAB communities from the different sampling points were analyzed for the number of operational taxonomic units (OTUs), species richness, and biodiversity using the QIIME program. The unweighted UniFrac distance matrices were constructed from the phylogenetic tree and used to conduct the principal coordinate analyses (PCoA). The obtained DNA sequences were deposited in the DDBJ sequence read archive (Accession Number: DRA002414).

## Additional Information

**How to cite this article**: Liu, G. *et al.* Comparison of Particle-Associated Bacteria from a Drinking Water Treatment Plant and Distribution Reservoirs with Different Water Sources. *Sci. Rep.* **6**, 20367; doi: 10.1038/srep20367 (2016).

## Supplementary Material

Supplementary Information

## References

[b1] Proctor C. R. & Hammes F. Drinking water microbiology—from measurement to management. Curr. Opin. Biotech. 33, 87–94 (2015). 25578740 10.1016/j.copbio.2014.12.014

[b2] Liu G., Verberk J. Q. J. C. & Dijk J. C. Bacteriology of drinking water distribution systems: an integral and multidimensional review. Appl. Microbiol. Biotechnol. 97, 9265–9276 (2013). 24068335 10.1007/s00253-013-5217-y

[b3] Liu G. *et al.* Quantification and identification of particle associated bacteria in unchlorined drinking water from three treatment plants by cultivation-independent methods. Water Res. 47, 3523–3533 (2013). 23618316 10.1016/j.watres.2013.03.058

[b4] Gauthier V., Barbeau B., Millette R., Block J. C. & Prévost M. Suspended particles in the drinking water of two distribution systems. Wa. Sci. Technol. 1, 237–245 (2001).

[b5] Matsui Y., Yamagishi T., Terada Y., Matsushita T. & Inoue T. Suspended particles and their characteristics in water mains: Developments of sampling methods. J. Water Supply Res. T. 56, 13–24 (2007).

[b6] Vreeburg J. H. G. & Boxall D. J. B. Discolouration in potable water distribution systems: A review. Water Res. 41, 519–529 (2007). 17174377 10.1016/j.watres.2006.09.028

[b7] Lehtola M. J., Nissinen T. K., Miettinen I. T., Martikainen P. J. & Vartiainen T. Removal of soft deposits from the distribution system improves the drinking water quality. Water Res. 38, 601–610 (2004). 14723929 10.1016/j.watres.2003.10.054

[b8] Gauthier V., Gérard B., Portal J. M., Block J. C. & Gatel D. Organic matter as loose deposits in a drinking water distribution system. Water Res. 33, 1014–1026 (1999).

[b9] Liu G., Lut M. C., Verberk J. Q. J. C. & Van Dijk J. C. A comparison of additional treatment processes to limit particle accumulation and microbial growth during drinking water distribution. Water Res. 47, 2719–2728 (2013). 23510692 10.1016/j.watres.2013.02.035

[b10] Torvinen E. *et al.* Mycobacteria in water and loose deposits of drinking water distribution systems in Finland. Appl. Environ. Microbiol. 70, 1973–1981 (2004). 15066787 10.1128/AEM.70.4.1973-1981.2004 PMC383162

[b11] Boe-Hansen R., Albrechtsen H. J., Arvin E. & Jørgensen C. Bulk water phase and biofilm growth in drinking water at low nutrient conditions. Water Res. 36, 4477–4486 (2002). 12418650 10.1016/s0043-1354(02)00191-4

[b12] Van Thienen P., Vreeburg J. & Blokker E. Radial transport processes as a precursor to particle deposition in drinking water distribution systems. Water Res. 45, 1807–1817 (2011). 21186040 10.1016/j.watres.2010.11.034

[b13] Vreeburg J. H. G., Schippers D., Verberk J. Q. J. C. & van Dijk J. C. Impact of particles on sediment accumulation in a drinking water distribution system. Water Res. 42, 4233–4242 (2008). 18789809 10.1016/j.watres.2008.05.024

[b14] Liu G. *et al.* Pyrosequencing reveals bacterial communities in unchlorinated drinking water distribution system: an integral study of bulk water, suspended solids, loose deposits, and pipe wall biofilm. Environ Sci Technol 48, 5467–5476 (2014). 24766451 10.1021/es5009467

[b15] Brazos B. J. & O’Connor J. T. Seasonal effects on generation of particle-associated bacteria during distribution. J. Environ. Eng. 122, 1050–1057 (1996).

[b16] Wojcicka L., Baxter C. & Hofmann R. Impact of particulate matter on distribution system disinfection efficacy. Water Qual. Res. J. Can. 43, 55–62 (2008).

[b17] Camper A. K., LeChevallier M. W., Broadaway S. C. & McFeters G. A. Bacteria associated with granular activated carbon particles in drinking water. Appl. Environ. Microbiol. 52, 434–438 (1986). 3767356 10.1128/aem.52.3.434-438.1986 PMC203552

[b18] Dietrich J. P., Loge F. J., Ginn T. R. & Başağaoğlu H. Inactivation of particle-associated microorganisms in wastewater disinfection: modeling of ozone and chlorine reactive diffusive transport in polydispersed suspensions. Water Res. 41, 2189–2201 (2007). 17389144 10.1016/j.watres.2007.01.038

[b19] Herson D. S., Marshall D. R., Baker K. H. & Victoreen H. T. Association of microorganisms with surfaces in distribution systems. J Am Water Works Ass. 83, 103–106 (1991).

[b20] Echeverría F. *et al.* Characterization of deposits formed in a water distribution system. Caracterización de depósitos formados en un sistema de distribución de agua potable 17, 275–281 (2009).

[b21] Verberk J. Q. J. C., Hamilton L. A., O’Halloran K. J., Van Der Horst W. & Vreeburg J. Analysis of particle numbers, size and composition in drinking water transportation pipelines: results of online measurements. Wa. Sci. Technol. 6, 35–43 (2006).

[b22] Prest E. *et al.* Combining flow cytometry and 16S rRNA gene pyrosequencing: a promising approach for drinking water monitoring and characterization. Water Res. 63, 179–189 (2014). 25000200 10.1016/j.watres.2014.06.020

[b23] Lautenschlager K. *et al.* A microbiology-based multi-parametric approach towards assessing biological stability in drinking water distribution networks. Water Res. 47, 3015–3025 (2013). 23557697 10.1016/j.watres.2013.03.002

[b24] Pinto A. J., Schroeder J., Lunn M., Sloan W. & Raskin L. Spatial-temporal survey and occupancy-abundance modeling to predict bacterial community dynamics in the drinking water microbiome. mBio 5, e01135–14 (2014). 24865557 10.1128/mBio.01135-14 PMC4045074

[b25] Hong P. Y. *et al.* Pyrosequencing analysis of bacterial biofilm communities in water meters of a drinking water distribution system. Appl. Environ. Microbiol. 76, 5631–5635 (2010). 20581188 10.1128/AEM.00281-10 PMC2918972

[b26] Glöckner J. *et al.* Phylogenetic diversity and metagenomics of candidate division OP3. Environ. Microbiol. 12, 1218–1229 (2010). 20158507 10.1111/j.1462-2920.2010.02164.x

[b27] Sun H., Shi B., Bai Y. & Wang D. Bacterial community of biofilms developed under different water supply conditions in a distribution system. Sci. Total. Environ. 472, 99–107 (2014). 24291134 10.1016/j.scitotenv.2013.11.017

[b28] Winkelmann N. & Harder J. An improved isolation method for attached-living Planctomycetes of the genus Rhodopirellula. J. Microbiol. Meth. 77, 276–284 (2009). 10.1016/j.mimet.2009.03.002 19303037

[b29] Berg J. M., Tymoczko J. L. & Stryer L. Biochemistry 5th edn, Section 2.4, (New York, 2002).

[b30] Jordaan K. & Bezuidenhout C. C. The impact of physico-chemical water quality parameters on bacterial diversity in the Vaal River, South Africa. Water SA 39, 385–396 (2013).

[b31] Magic-Knezev A. & van der Kooij D. Optimisation and significance of ATP analysis for measuring active biomass in granular activated carbon filters used in water treatment. Water Res. 38, 3971–3979 (2004). 15380987 10.1016/j.watres.2004.06.017

[b32] Hammes F. *et al.* Flow-cytometric total bacterial cell counts as a descriptive microbiological parameter for drinking water treatment processes. Water Res. 42, 269–277 (2008). 17659762 10.1016/j.watres.2007.07.009

[b33] Hwang C., Ling F., Andersen G. L., Lechevallier M. W. & Liu W. T. Evaluation of methods for the extraction of DNA from drinking water distribution system biofilms. Microbes Environ. 27, 9–18 (2011). 22075624 10.1264/jsme2.ME11132 PMC4036026

[b34] Tamaki H. *et al.* Analysis of 16S rRNA amplicon sequencing options on the roche/454 next-generation titanium sequencing platform. PLoS ONE. 6, e25263 (2011). 21966473 10.1371/journal.pone.0025263 PMC3179495

