# Supplementary material for: Comparison of Particle-Associated Bacteria from a Drinking Water Treatment Plant and Distribution Reservoirs with Different Water Sources
Source: Sci Rep. 2016 Feb 2;6:20367. doi: 10.1038/srep20367 (PMC4735813; doi:10.1038/srep20367)
Supplement: Supplementary Information [file srep20367-s1.doc]

**Support Information for:**

Comparison of Particle-Associated Bacteria from Drinking Water Treatment Plant and Distribution Reservoirs with Different Water Sources

G. Liu1,2*, F.Q. Ling­3, E.J. van der Mark4, X.D. Zhang1, A. Magic-Knezev5, J.Q.J.C. Verberk1, W.G.J. van der Meer1,2, G.J. Medema1,6, W.T. Liu3, J.C. van Dijk1

1 Sanitary engineering, Department of Water Management, Faculty of Civil Engineering and Geosciences, Delft University of Technology, P.O. Box 5048, 2600 GA Delft, the Netherlands. E-mail: [G.liu-1@tudelft.nl](mailto:G.liu-1@tudelft.nl) / [ganghow@gmail.com](mailto:ganghow@gmail.com)

2 Oasen Water Company, PO BOX 122, 2800 AC, Gouda, the Netherlands

3 Department of Civil and Environmental Engineering, University of Illinois Urbana-Champaign, 205 N. Mathews Ave., Urbana, Illinois 61801, U.S.A.

4 Dunea Water company, P.O. Box 756, 2700 AT Zoetermeer, the Netherlands

5 Het Water Laboratorium, P.O. Box 734, 2003 RS Haarlem, the Netherlands

6 KWR Watercycle Research Institute, P.O. Box 1072, 3430 BB Nieuwegein, The Netherlands

Summary: 8 pages with 3 tables and 7 figures are included below.

Table S1 General information about the waters before and after mixing (average ± std., n=3.)

|  | ARR-TP | ARR-D | Mixed | SW-D |
| --- | --- | --- | --- | --- |
| TCC  (cells ml-1) | 0.5(±0.1)×105 | 1.1(±0.1)×105 | 1.6(±0.3)×105 | 2(±0.2)×105 |
| ATP  (ng l-1) | 1.6 (±0.1) | 2.1 (±0.2) | 3.1 (±0.6) | 4.5 (±0.2) |
| TP  (µg l-1) | 50 (±2) | 40 (±4) | 20 (±2) | 10 (±3) |
| DOC  (mg l-1) | 2.1 (±0.3) | 2.1 (±0.3) | 1.9 (±0.7) | 1.4 (±0.3) |
| Particles  (# ml-1, measured online) | 20 (±3) | 25 (±5) | 230 (±17) | 90 (±11) |
| A-TCC/particle  average cells per particle | 48 (±10) | 91 (±9) | 16 (±2) | 7 (±2) |

In this table, TCC is the total cell count, ATP is adenosine triphosphate, TP is the total phosphate, DOC is the dissolved organic carbon, and A-TCC is the attached cell number. The water from the ARR-TP and the SW-D are characterized as biologically stable based on the assimilable organic carbon (AOC) results.

Table S2 The PAB diversity in the ARR water, the surface water and the water at mixing reservoir in the DWDS (average ± std., n=3.)

| Sample | Sequences Before Denoising | Sequences After Denoising | Observed OTUs (97%) | Chao 1 (97%) | Shannon Index (H) |
| --- | --- | --- | --- | --- | --- |
| ARR-TP | 3092 | 2988 | 483 ± 13 | 1361 ± 56 | 8.0 ± 0.1 |
| ARR-D | 6647 | 6377 | 1112 ± 11 | 2597 ± 94 | 8.5 ± 0.1 |
| Mixed | 23713 | 22496 | 1285 ± 45 | 4073 ± 200 | 9.2 ± 0.2 |
| SW-D | 10670 | 10423 | 864 ± 21 | 1534 ± 122 | 8.4 ± 0.1 |

Table S3 The genera identified in the PAB at each sampling location

(>1% at least one sampling location)

| Genera | Percentage of sequences (%) | | | |
| --- | --- | --- | --- | --- |
|  | ARR-TP | ARR-D | Mixed | SW-D |
| *Limnobacter* | 0 | 0 | 2.4 | 4.2 |
| *Caldilinea* | 0 | 0 | 0.5 | 3.2 |
| *CandidatusOdyssell* | 0 | 0 | 0.4 | 1.8 |
| *Unclassified genus of Oxalobacteraceae* | 0 | 0 | 1.3 | 4.3 |
| *Rhodopirellula* | 0 | 0 | 0.7 | 1.9 |
| *Gallionella* | 0 | 0.3 | 1.0 | 0.3 |
| *Polynucleobacter* | 0 | 0.1 | 5.3 | 0.2 |
| Unclassified genus of *Methanosarcinaceae* | 1.3 | 1.8 | 0.4 | 0 |
| *LCP-6* | 1.9 | 2.1 | 0.6 | 0 |
| *KPJ58RC (OP3)* | 4.3 | 5.1 | 3.0 | 0 |
| *GIF10 (OP3)* | 7.8 | 8.0 | 4.8 | 0 |
| *Koll11 (OP3)* | 1.4 | 1.7 | 1.2 | 0 |
| *BD4-9 (OP3)* | 2.7 | 3.2 | 2.1 | 0 |
| *PBS-25 (OP3)* | 1.0 | 1.3 | 1.5 | 0 |
| *Unclassified genus of Rhodoplanes* | 0.3 | 0.4 | 1.1 | 2.3 |
| *MIZ46 (Deltaproteobacteria)* | 0.2 | 0.3 | 0.9 | 1.2 |
| *Aquabacterium* | 3.2 | 0.2 | 0.2 | 0.7 |
| *Syntrophobacteraceae* | 4.4 | 3.0 | 1.8 | 0.9 |
| *Planctomyces* | 1.0 | 0.9 | 2.1 | 5.3 |
| *Rhodocyclales* | 1.5 | 1.5 | 1.3 | 1.6 |
| Unclassified genus of *Comamonadeaceae* | 4.4 | 1.7 | 14.7 | 6.9 |
| *Legionella* | 0.3 | 0.6 | 1.0 | 0.8 |
| *Nitrospira* | 1.3 | 2.1 | 1.0 | 1.0 |
| *Crenothrix* | 0.9 | 1.9 | 9.4 | 8.0 |
| Unclassified genus in *Alphaproteobacteria* | 1.7 | 3.0 | 5.7 | 13.3 |
| Unclassified genus in unclassified Phyla | 22.2 | 22.7 | 14 | 10.5 |
| Other | 38.2 | 38.1 | 21.6 | 31.6 |

A:
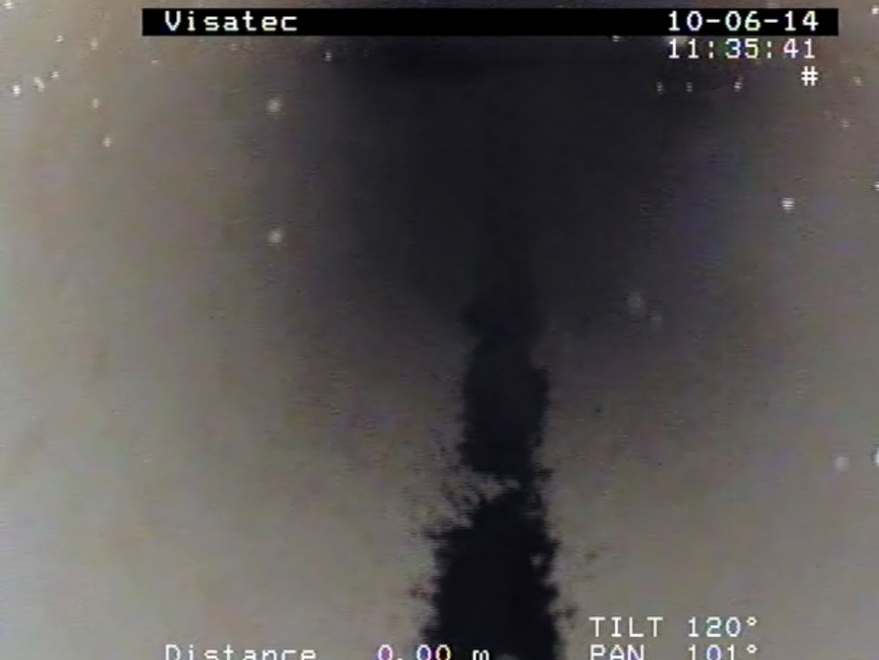


B:
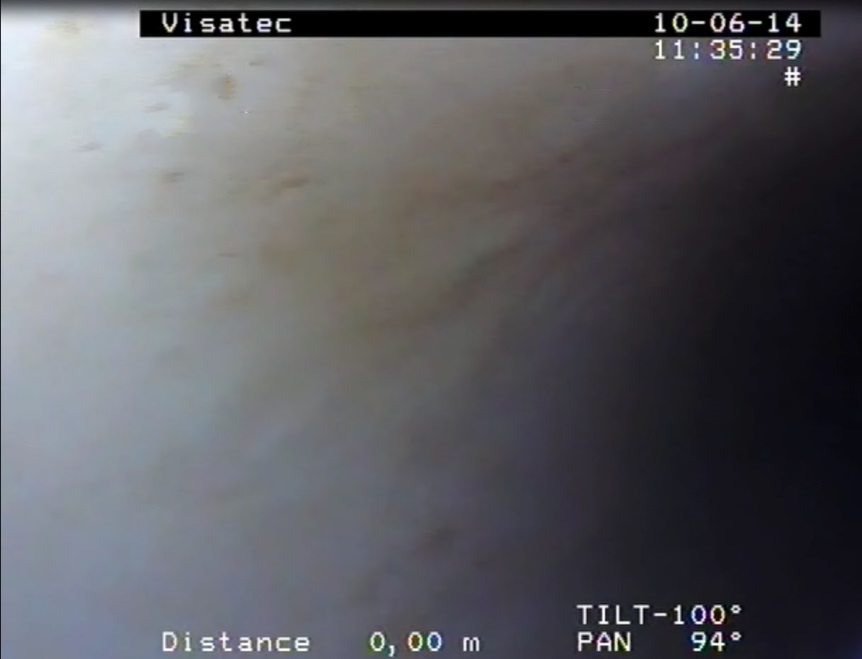


C:
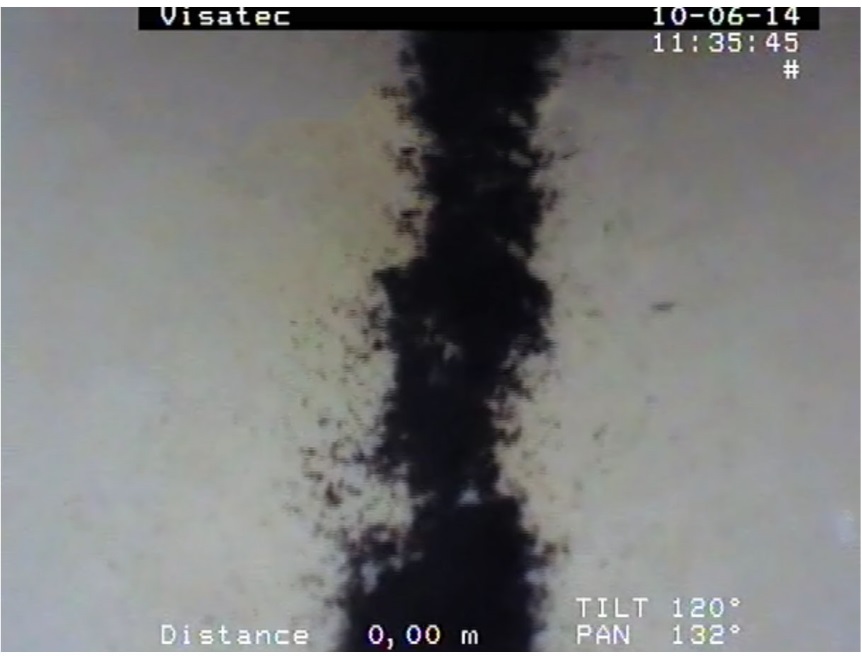


Figure S1. Pictures of inner pipe (D=110mm, PVC) taken from an distribution pipe under operation, illustrating diffferent phases present in distribution pipes. (A) gives an general view of the pipe, with the reflection dots showing the big suspended particles; (B) zoomed into the pipe surface, showing the pipe wall biofilm; (C) zoomed into the pipe bottom showing the loose deposits accumulated in the pipe. The four phases are: the bulk water, pipe-wall biofilm, suspended solids, and loose deposits. For our purposes, we defined loose deposits as the portion of those particles which accumulate in distribution pipes under normal flow condition that can be flushed out by increasing the flow to 1.5 m/s (standardized flushing event worldwide).

A:
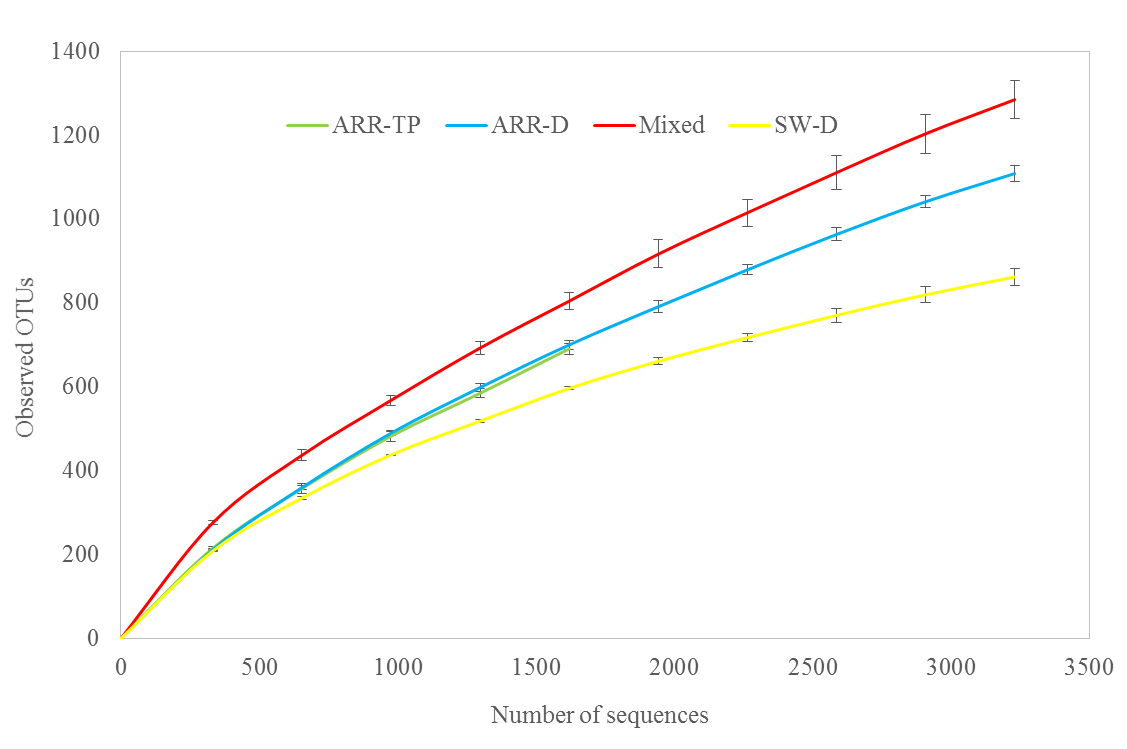


B:
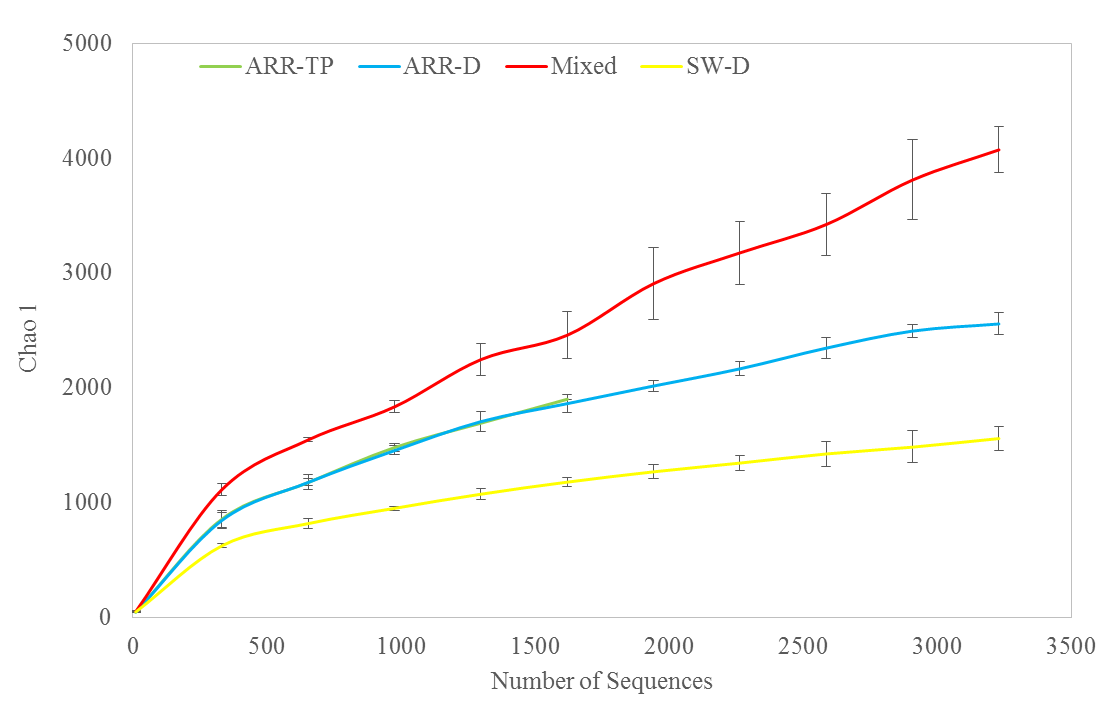


Figure S2. Rarefaction curves of observed OTUs (A) and Chao 1 (B).


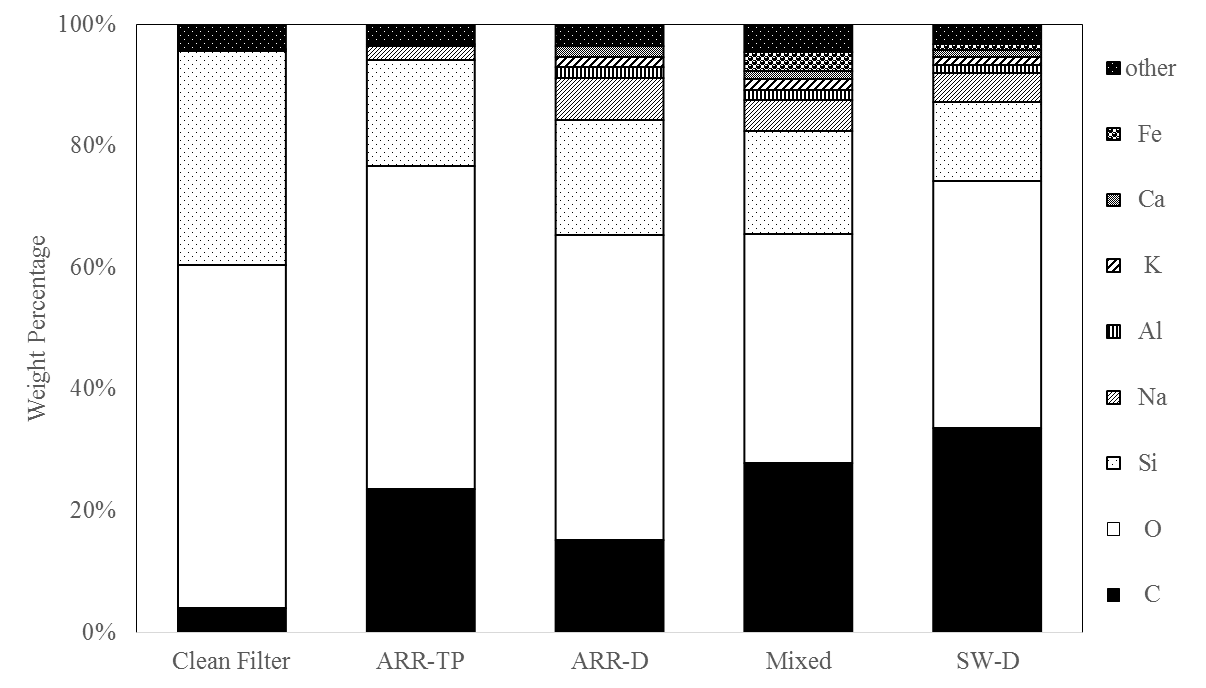


Figure S3. Elemental composition of the collected PAB analyzed by EDS.
